# A clinicopathological study and survival analysis of 99 breast cancers with HER2/CEP17 ratio ≥ 2.0 and an average HER2 copy number < 4.0 per cell in China

**DOI:** 10.1186/s12885-023-10531-z

**Published:** 2023-01-25

**Authors:** Shuling Zhou, Hong Lv, Anqi Li, Ming Li, Siyuan Zhong, Hongfen Lu, Xiaoyan Zhou, Qianming Bai, Wentao Yang

**Affiliations:** 1 Department of Pathology, Fudan University Shanghai Cancer Center, 270 Dong’an Road, Shanghai, 200032 P. R. China; 2 Department of Oncology, Shanghai Medical College, Fudan University, 270 Dong’an Road, Shanghai, 200032 P. R. China

**Keywords:** Breast cancer, HER2, Anti-HER2 therapy

## Abstract

**Background:**

Breast cancer patients of American Society of Clinical Oncology and the College of American Pathologists (ASCO/CAP) Group 2 were all HER2-negative according to the 2018 guideline, not HER2-positive as defined in the 2013 guideline.

**Methods:**

We aims to elucidate the unique clinicopathological features of ASCO/CAP Group 2 patients by comparing with classic HER2-nonamplified cancers, and reveal the efficacy of the former to anti-HER2 therapy. The clinicopathological features, treatment and prognosis information of 99 patients between 2014 and 2018 were collected. HER2 status was re-defined using the updated recommendations.

**Results:**

Of the 99 ASCO/CAP Group 2 tumors, 25.5% (25/99) tumors were immunohistochemical (IHC) 0/1+ and 74.7% (74/99) tumors were IHC 2+. According to the updated 2018 guideline, all of them were HER2 negative. When compared to ASCO/CAP Group 5, patients of ASCO/CAP Group 2 displayed higher ratio of histological grade 3 (*P = .*03), high Ki67 proliferation index (*P = .*03) and pN3 (more than 9 lymph nodes metastasis, *P = .*02), and lower estrogen receptor (ER) positivity (*P = .*04). There was no statistical difference in the survival of patients received anti-HER2 therapy and patients not received anti-HER2 therapy.

**Conclusions:**

Patients of ASCO/CAP Group 2 did not received apparent benefit from anti-HER2 treatment. Although according to the updated guidelines and latest reports, HER2 is negative, but when compared with classic HER2-nonamplified cancers, patients of this group seemed to be more aggressive. We suggest that this group still be regarded as an independent category, in order to accumulate more cases in the future to expand the scope of research.

**Supplementary Information:**

The online version contains supplementary material available at 10.1186/s12885-023-10531-z.

## Background

The gene for human epidermal growth factor receptor 2 (HER2) is located on the long arm of chromosome 17 and encodes a transmembrane growth factor receptor with tyrosine kinase activity. Overexpression or amplification of HER2 occurs in approximately 15–20% of invasive breast cancers and is associated with poor prognosis [1–3]. As a well-established therapeutic target, HER2-directed therapies, such as trastuzumab, pertuzumab, and other anti-HER2 agents, have dramatically improved breast cancer-specific outcomes in HER2-positive breast cancers. However, the corresponding significant costs and potential toxicities cannot be ignored [4]. Therefore, it is critical to accurately assess HER2 status of breast cancers, and to correctly identify patients who might benefit from targeted therapy, while sparing patients who would not.

Immunohistochemical (IHC) analysis and dual-probe fluorescence in situ hybridization (FISH) are the two assays commonly used to test HER2 status in breast cancer specimens. The American Society of Clinical Oncology and College of American Pathologists (ASCO /CAP) have periodically issued detailed guidelines and updates for conducting and interpreting HER2 tests.

The 2013 guidelines determine breast cancers with a FISH dual-probe HER2/CEP17 ratio of 2 or greater as amplified, irrespective of HER2 copy number [4]. Consequently, breast cancer with average HER2 signal < 4.0 per cell and HER2/CEP17 ratio ≥ 2.0 are reported as HER2 positive. Based on the subsequent studies, the updated 2018 HER2 testing guidelines do not report this category as HER2 positive directly, but call for additional work-up [5] and the final HER2 status would be highly depend on IHC results. According to subsequent published works, breast cancer of this category, different from the classic biologically HER2-positive cases, are predominantly HER2 negative by IHC [6, 7]. As a result, breast cancers with average HER2 signal < 4.0 per cell and HER2/CEP17 ratio ≥ 2.0 will be mostly classified as HER2 negative (without amplification) finally.

This pattern often occurs with low average number of HER2 copies (< 4) and loss of chromosome 17 copy number due to true monosomy (loss of a chromosome 17), loss of portion of chromosome 17, or genetic alterations that impair the CEP17 binding [8]. ASCO/CAP Group 2 tumors with monosomy of chromosome 17 (m17) have not been studied extensively. There is still debate over the features of breast cancer with m17, with some arguing for a worse outcome and less responsiveness to anti-HER2 targeted therapy [9]. The purpose of this study is to explore the clinicopathological profile of breast cancers of ASCO/CAP Group 2 and to provide information for clinical management. By comparing them with classic HER2 negative group (ASCO/CAP Group 5, average HER2 signal < 4.0 per cell and HER2/CEP17 ratio < 2.0), we hope to provide some insights into their actual similarity to HER2-negetive breast cancers. For the sake of simplicity, in the following, ASCO/CAP Group 2 and ASCO/CAP Group 5 were denoted as Group 2 and Group 5, respectively.

## Materials and methods

### Sample collection

After obtaining approval from the local Institutional Review Boards and the local research ethics committees of the authors’ institutions, breast cancers in the Department of Pathology in Fudan University Shanghai Cancer Center between 2014 and 2018 were retrieved and reviewed.

Cases that meet the following criteria were enrolled: 1. HER2 status had been tested by both IHC and FISH; 2. Both in-house and consultation invasive breast cancer with average HER2 signal < 4.0 per cell and HER2/CEP17 ratio ≥ 2.0 (Group 2), including primary, metastatic and recurrent cases; 3. In-house and primary invasive breast cancer with modified radical surgery and no neoadjuvant therapy prior to surgery, and with average HER2 signal < 4.0 per cell and HER2/CEP17 ratio < 2.0 (Group 5). Totally, there were 40,201 in-house patients of breast cancers from 2014 to 2018. A total of 99 cases of Group 2 and randomly screened 374 cases of Group 5 were enrolled finally. Group 2 consists of 67 in-house cases and 32 consultation cases. Clinical and pathological features, including patient’s age, histological subtype, grade, tumor size, lymph node status, treatments and follow-up information were obtained from patient clinical histories and pathology reports. The IHC and FISH slides of HER2 testing were re-evaluated by two breast pathologists separately. Tumor stage of primary invasive breast cancer without preoperative treatment was evaluated according to the 8th edition of the American Joint Committee on Cancer (AJCC) staging system [10].

### Morphology observation

The surgical specimens of 441 in-house tumors, including 67 cases of Group 2 and 374 cases of Group 5, were routinely processed and sectioned into 4-μm sections and stained with hematoxylin-eosin. All of the 32 consultation tumors were submitted as HE slides with either representative paraffin blocks or unstained slides. The tumors examined in this study were reviewed by two breast pathologists separately. The histological type was characterized based on the World Health Organization histologic classification of breast tumors (2019 version) [11]. A modified Bloom and Richardson score scheme [12] was used to determine the histological grade of invasive carcinomas. Morphological features such as histological subtype, tumor grade, lymph node status were all observed. Six patients of metastatic or recurrent breast cancer in Group 2 were excluded prior to grading and AJCC staging. Pathological responses to neoadjuvant chemotherapy were evaluated by the systems of Miller-Payne (MP). MP Grade 1–4 are categorized as partial pathological response (pPR) and MP grade 5 as pathological complete response (pCR) [13].

### Immunohistochemical procedures and evaluation

All 473 cases were immunohistochemically assessed for estrogen receptor (ER), progesterone receptor (PR), HER2, and Ki67. All of the antibodies used in this study were from Roche Ventana. The staining was performed with the Ventana BenchMark ultra autostainer (Ventana Medical System Inc., Roche, Tuscon, AZ, USA) and the Ventana Ultra View Universal DAB detection kit. The scoring criterion from the ASCO/CAP [5] was used to reevaluate HER2 status. Staining was considered positive for ER and PR when the nuclear staining was observed in 1% or more of the tumor cells [14]. The Ki67 labeling index was determined by counting the number of Ki67-positive cancer nuclei from a total of 1000 cancer cells. Ki67 values of 30% or above could be considered as high proliferation [15].

According to the 2013 St Gallen recommendations [15], all tumors were further classified into the following immunohistochemical surrogate subtypes. Luminal A-like subtype: ER positive, PR positive, HER2 negative and Ki-67 low. Luminal B-like HER2 negative subtype: ER positive, HER2 negative, with Ki-67 high or PR negative or low; Luminal B-like HER2 positive subtype: ER positive, HER2 positive with any PR or Ki-67. HER2 overexpression subtype: HER2 gene amplification or protein over-expression, with both ER and PR negative. Triple negative subtype (TNBC): both hormonal receptors and HER2 negative. The cut-off point of Ki67 index and PR low was set to 20% according to the recommendation of 2013 St. Gallen International Expert Consensus.

### Fluorescence in situ hybridization (FISH)

Formalin fixed paraffin-embedded tissue specimens were sectioned into 4-μm slides for HER2 FISH testing. Hematoxylin and eosin (H&E) stained sections were evaluated to label the invasive carcinoma. FISH analyses were performed using the FDA approved PathVysion HER2 DNA probe kit (Abbott Molecular, Des Plaines, IL, USA). HER2 and CEP17 signals were manually counted and analyzed independently by two certified pathologists. Each person individually counted 30 cells from two non-overlapping areas and calculated the corresponding HER2/CEP17 ratios. If their counts are comparable, an average was used to determine the final copy numbers; otherwise, the counts would be repeated. Chromosome 17 monosomy (m17) is defined as average CEP17 signal per nucleus less than 1.4 [16].

### Statistical analysis

Descriptive statistics of the clinicopathologic features of the 473 tumors were calculated. All cases of Group 2 were grouped for different cutoffs of average HER2 signals, average CEP17 signals, and HER2/CEP17 ratio, respectively. Various tumor characteristics were compared between these groups using the Pearson chi-square test aiming at evaluating the relationship between different groups. A total of 57 patients of Group 2 who were primary breast cancer without neoadjuvant therapy and underwent modified radical mastectomy were selected and matched with 374 patients of Group 5 for further comparison analysis of clinicalpathological characteristics. The Fisher exact tests were performed when necessary. All statistical tests were two-sided, and *P* values less than 0.05 were considered as significant. Disease free survival (DFS) was the primary end point, defined as local or regional recurrence, distant metastasis, or death from any cause. DFS and overall survival (OS) of Group 2 were estimated using the Kaplan-Meier method. All analyses were performed in SPSS (version 17.0, SPSS Company, Chicago, IL).

## Results

### Clinical-pathological features

All 473 patients from group 2 and group 5 were female with mean and median age of 52y, 45y, respectively (range 24-79y). The median age at diagnosis was 56 years (range 24 to 79y) for Group 2, and 52 years (range 26 to 80y) for Group 5. The clinicalpathological characteristics of the 99 patients of Group 2 were summarized in Table 1.

**Table 1:** Clinico-pathological characteristics of 99 patients of ASCO/CAP Group 2

Characteristics	Value (%)
Primary breast cancer	93 (93.9%)
With neoadjuvant therapy	12 (12.9%)
Without neoadjuvant therapy	81 (87.1%)
Recurrent or metastatic breast cancer	6 (0.6%)
Metastasis	4 (66.7%)
Recurrence	2 (33.3%)
Age (range 24-79y,median 56y) ^a^	
≤ 50	43 (46.2%)
> 50	50 (53.8)
Histological subtype ^b^	
ICONST	77 (95.1%)
Mixed ICONST and invasive micropapillary carcinoma	1 (1.2%)
Mixed ICONST and mucinous carcinoma	1 (1.2%)
Matrix producing carcinoma	1 (1.2%)
Invasive carcinoma with neuroendocrine differentiation	1 (1.2%)
Histological grade ^b^	
G2	28 (34.6%)
G3	53 (65.4%)
Pathologocal stage ^b^	
T1	37 (45.7%)
T2	34 (42.0)%
T3	3 (3.7%)
Non-available	7 (8.6%)
Lymph node stage ^b^	
pN0	39 (48.1%)
pN1	21 (25.9%)
pN2	13 (16.0%)
pN3	4 (4.9%)
Non-available	4 (4.9%)
AJCC staging ^b^	
I	25 (30.9%)
II	32 (39.5%)
III	15 (18.5%)
Non-available	9 (11.1%)
ER ^a^	
Positive	73 (78.5%)
Negative	20 (21.5%)
PR ^a^	
Positive	64 (68.8%)
Negative	29 (31.2%)
HER2-IHC ^a^	
Negative (0,1+)	25 (26.9%)
Equivocal (2+)	68 (73.1%)
Ki67 ^a^	
> 30%	43 (46.2%)
≤ 30%	50 (53.8%)
Immunohistochemical surrogate subtype ^a^	
Lumina A-Like	25 (26.9%)
Lumina B-Like	48 (51.6%)
TNBC	20 (21.5%)
Surgery ^b^	
Modified radical mastectomy	57 (70.4%)
Simple mastectomy +Sentinel lymph node biopsy	12 (14.8%)
Conservative surgery +Sentinel lymph node biopsy	8 (9.9%)
Non-available	4 (4.9%)
anti-HER2 therapy ^a^	
Yes	48 (51.%)
No	43 (46.2%)
Non-available	2 (2.2%)

^a^Primary breast cancer with neoadjuvant therapy

^b^Primary breast cancer without neoadjuvant therapy

**Fig. 1:**
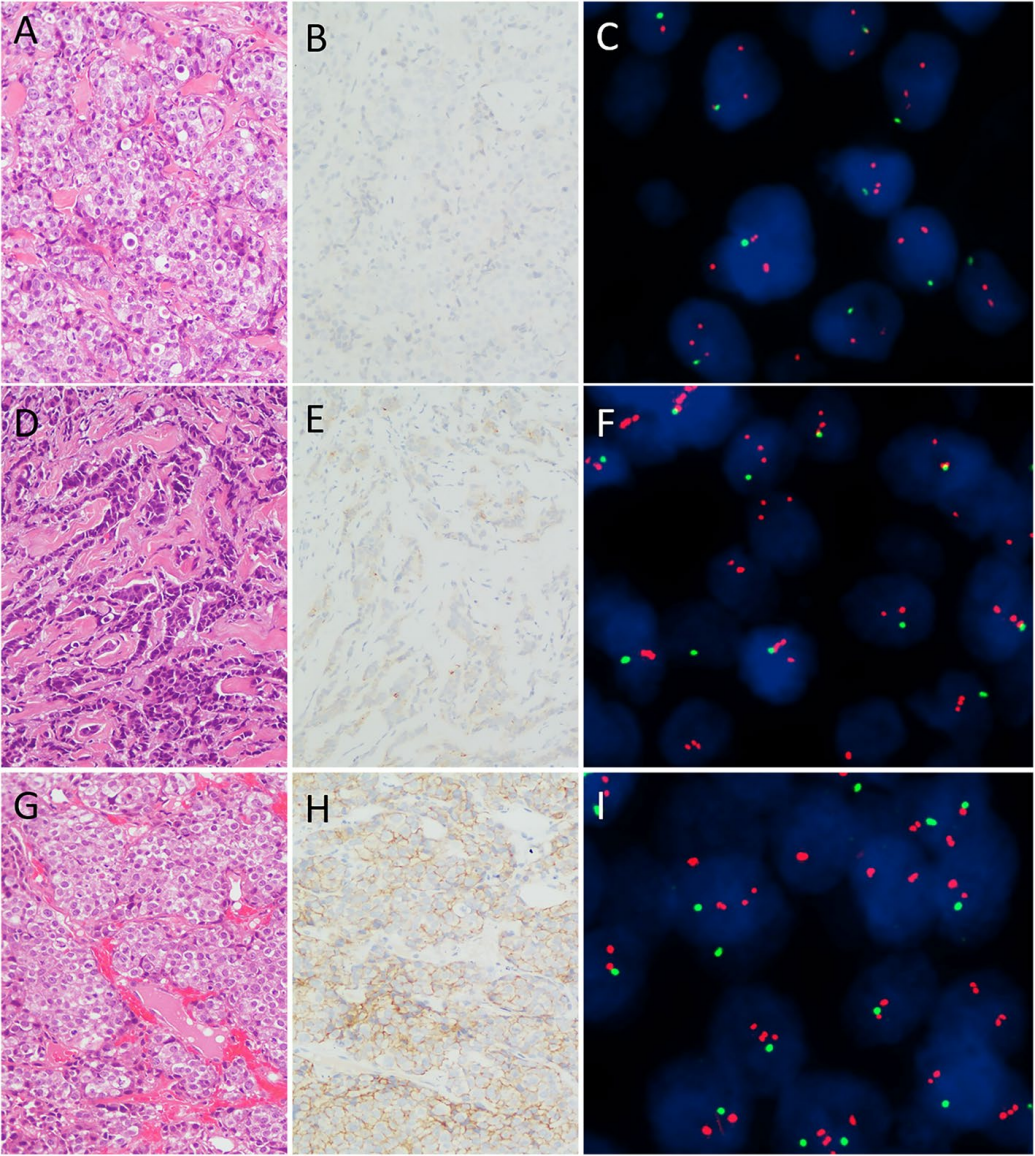
Example of invasive carcinoma of no special type with HER2 immunostaining 0, 1+, and 2+ and corresponding fluorescence in-situ hybridization images. **A**, **D**, **G**, H&E-stained section of invasive carcinoma of no special type (original magnification ×20). **B**, **E**, **H** invasive carcinoma of no special type displayed HER2 IHC 0，1+ and 2+, respectively (original magnification ×20). **C** fluorescence in-situ hybridization image corresponding to **A** and **B** exhibited normal HER2 signals with HER2 (red)/CEP17 (green) ratio of 2.7 and average HER2 signal of 2.5. **F** fluorescence in-situ hybridization image corresponding to **D** and **E** displayed HER2 (red)/CEP17 (green) ratio of 4.5 and average HER2 signal of 2.5. **I** fluorescence in-situ hybridization image corresponding to **G** and **H** displayed HER2 (red)/CEP17 (green) ratio of 2.8 and average HER2 signal of 2.8

Most of the 81 primary breast cancer patients of Group 2 (95.1%, 77/81) were invasive carcinoma of no special type (ICONST) (Table 1, Fig. 1A, D and G). The histological subtype of Group 5 consisted of 370 (98.9%, 370/374) ICONST, 3 (0.8%, 3/374) invasive micropapillary carcinoma, and 1 (0.3%, 1/374) invasive lobular carcinoma. According to the Nottingham modification of the Bloom-Richardson grading system [11], 1.6% (6/374), 55.6% (208/374) and 42.8% (160/374) of the Group 5 tumors were histological grade 1, grade 2, and grade 3, respectively.

The available information including the clinico-pathological characteristics, molecular findings and follow up information of 12 patients of Group 2 who received neoadjuvant therapy prior to surgery were summarized in Table 2. Two of them showed pCR with MP grade 5, and five of pPR consisting of one MP grade 4 and four MP grade 3. The MP grade of the other 5 patients of Group 2 was unknown, because three of them died during neoadjuvant therapy and the other two cannot be contacted anymore.

**Table 2:** Clinico-pathological parameters, Miller-Payne grade and follow up information of 12 ASCO/CAP group 2 patients who underwent neoadjuvant therapy prior to surgery

Case	Age	MP grade	Histological subtype	Histological grade	Diameter(cm)	ALN	HER2 IHC	HER2 status according to 2018 ASCO/CAP	Immunohistochemical surrogate subtype	Anti-HER2 therapy	Disease progresions	Follow up
1	39	NA	NA	NA	NA	NA	1	non-amplification	TNBC	Y	Y, metastasis before anti-HER2 therapy	survive,39 months
2	43	3	ICONST	2	2	0/15	1	non-amplification	Luminal A	Y	N	survive,38 months
3	52	NA	NA	NA	NA	NA	1	non-amplification	Luminal B	N	N	survive,39 months
4	49	3	invaisive carcinoma with neuroendocrine differentiation	2	1.5	0/8	2	non-amplification	Luminal A	N	N	survive,44 months
5	57	NA	NA	NA	NA	NA	2	non-amplification	Luminal B	N	Y,16 months	die,16 months
6	41	5	/	/	/	0/19	1	non-amplification	TNBC	Y	N	survive,29 months
7	39	3	ICONST	2	1.7	1/18	2	non-amplification	Luminal B	Y	N	survive,24 months
8	63	3	ICONST	2	3.6	3/14	1	non-amplification	Luminal B	Y	N	survive,24 months
9	52	NA	NA	NA	NA	NA	2	non-amplification	Luminal B	Y	Y, metastasis before anti-HER2 therapy	die,20 months
10	50	4	ICONST	/	/	0/13	2	non-amplification	TNBC	Y	N	survive,20 months
11	36	5	/	/	/	0/10	1	non-amplification	TNBC	N	N	survive,41 months
12	68	NA	NA	NA	NA	NA	2	non-amplification	TNBC	N	Y,16 months	die,16 months

*ALN* Axillary lymph node

*NA* Non-available

### HER2 status determination by IHC and FISH

HER2 was negative (IHC 0, IHC 1+, Fig. 1B and E) in 25 (25.3%, 25/99) Group 2 cases. The other 74 (74.7%, 74/99) Group 2 cases including 68 of primary breast cancer, 4 of metastatic breast cancers, and 2 of recurrence of breast cancer, showed equivocal HER2 immunostaining (IHC 2+, Fig. 1H). Within the Group 5 cases, 29 (7.8%, 29/374) showed HER2 negative, and 345 (92.2%, 345/374) exhibited equivocal HER2 immunostaining.

**Fig. 2:**
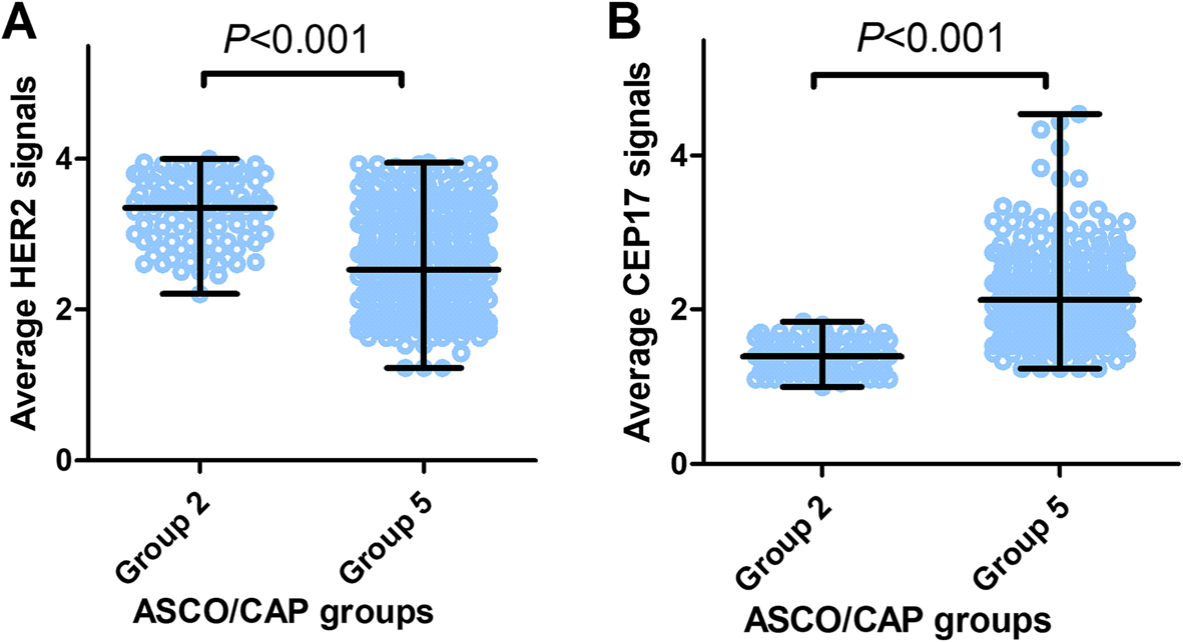
Average HER2 signals and average CEP17 signals of two ASCO/CAP groups. The average HER2 copy number of ASCO/CAP group 2 was significantly higher than that of ASCO/CAP group 5 (*p* < 0.0001, **A**), while the average CEP17 copy number was significantly lower than ASCO/CAP group 5 (*p* < 0.0001, **B**)

FISH detection showed that all 99 tumors belonged to Group 2 (Fig. 1C, F, I). The average HER2 signals per cell of Group 2 ranged from 2.2 to 4.0 with a mean and median of 3.3. Those of Group 5 ranged from 1.23 to 3.95 with a mean of 2.6 and a median of 2.18. The average HER2 signals were rather higher in Group 2 than in Group 5 (*p* < 0.0001) (Fig. 2A). The average CEP17 signals per cell of Group 2 ranged from 1.0 to 1.84 with a mean of 1.39 and a median of 1.4. In contrast, those of Group 5 ranged from 1.24 to 4.54 with a mean of 2.11 and a median of 1.94. The average CEP17 signals per cell were rather lower in Group 2 than in Group 5 (*p* < 0.0001) (Fig. 2B). The proportion of m17 is much higher in Group 2(50.5%, 50/99) than in Group 5(2.9%, 11/374) (*p* < 0.0001). In Group 2, the mean and median ratio of HER2/CEP17 was 2.37 and 2.3 respectively (range 2 to 3.41). In Group 5, the mean and median ratio of HER2/CEP17 was 1.25 and 1.1 respectively (range 0.4 to 1.94).

None of the Group 2 tumors were HER2 IHC 3+. According to the guideline of 2018 ASCO/CAP, all Group 2 tumors were determined as HER2 negative. There is no doubt that 374 tumors of the classic negative group (Group 5) were HER2 negative.

### Immunohistochemical surrogate subtype based on IHC and FISH

ER was positive in 78.5% (73/93) of the primary breast cancers of Group 2 (Table 1) and in 87.2% (326/374) cases of Group 5 (Table 3), respectively. The positive rate of PR of Group 2 and Group 5 was 68.8% (64/93) and 75.4% (282/374), respectively. The Ki67 index was high in 45.2% cases (42/93) of Group 2 and 30.7% (115/374) cases of Group 5. Most cases of Group 2 were of a luminal subtype (luminal A-like, 26.9%, 25/93; luminal B-like, 51.6%, 48/93), whereas the triple negative subtype accounted for 21.5% (20/93) of the primary breast cancers. Luminal subtype (luminal A-like, 28.6%, 107/374; luminal B-like, 59.1%, 221/374) was also the main subtype of Group 5. The proportion of triple negative subtype (12.3%, 46/374) was lower than that of Group 2. Since neither the Group 2 nor Group 5 HER2 genes were amplified, there was no HER2 overexpression subtype in either group.

**Table 3:** Comparison of clinico-pathologic features between 374 patients of ASCO/CAP group 5 and 57 primary breast cancer patients of ASCO/CAP group 2 who underwent modified radical mastectomy without neoadjuvant therapy

Characteristics	Group 2	Group 5	p
	HER2/CEP17 ≥ 2; HER2 copy<4 *n* = 57(%)	HER2/CEP17 < 2; HER2 copy<4 *n* = 374(%)	
Age (year)			0.73
≤ 50	27 (47.4)	168 (44.9)	
> 50	30 (52.6)	206 (55.1)	
Histologic grade			0.03
G1	0	6 (1.6)	
G2	22 (38.6)	208 (55.6)	0.03
G3	35 (61.4)	160 (42.8)	
Pathologic stage			0.79
T1	20 (39.2)	131 (35.0)	
T2	28 (54.9)	224 (59.9%)	
T3	3 (5.9)	19 (5.1)	
Lymph node status			0.02
pN0	20 (35.1)	150 (40.1)	
pN1	20 (35.1)	136 (36.4)	
pN2	13 (22.8)	84 (22.5)	
pN3	4 (7.0)	4 (1.1)	
AJCC staging			0.84
I	9 (17.6)	64 (17.1)	
II	27 (52.9)	213 (57.0)	
III	15 (29.4)	97 (25.9)	
ER			0.04
Positive	44 (77.2)	326 (87.2)	
Negative	13 (22.8)	48 (12.8)	
PR			0.16
Positive	38 (66.7)	282 (75.4)	
Negative	19 (33.3)	92 (24.6)	
Ki67			0.03
≥ 30%	26 (45.6)	115 (30.7)	
< 30%	31 (54.4)	259 (69.3)	
Immunohistochemical surrogate subtype			0.12
Lumina A-Like	18 (31.6)	107 (28.6)	
Lumina B-Like	27 (47.4)	221 (59.1)	
TNBC	12 (21.1)	46 (12.3)	

### Treatment and clinical follow-up information

The follow-up information of twelve patients of Group 2 who received neoadjuvant therapy prior to surgery was summarized in Table 2. Modified radical mastectomy was conducted in 7 (58.3%, 7/12) of the 12 patients after neoadjuvant therapy. Three patients died during neoadjuvant therapy without any surgery. Subsequent treatment after initial neoadjuvant therapy for two patients was unknown owing to loss of contact. Two (2/7, 28.6%) of the 7 patients that received modified radical mastectomy had metastases to axillary lymph nodes. Follow-up information was available for the 12 patients and 3 of them died. Two of the 3 patients died from lung metastasis 16 months after their initial diagnosis. The other one showed distant metastases at the time of initial diagnosis and survived for 20 months. None of the remaining 9 patients dies and the follow-up time ranged from 20 to 44 months, with a mean and median of 25 and 32 months. In addition to the above-mentioned distant metastasis in 3 patients, another one emerged upper supraclavicular lymph nodes and bone metastasis at the time of initial diagnosis. The other 8 patients survived without distant metastasis.

Eighty-one patients of Group 2 were primary breast cancer without preoperative treatment. Thirty-eight patients (46.8%, 38/81) had lymph node metastasis and 4 of them displayed more than 9 lymph nodes involvement, with a mean positive lymph node number of 15. Two patients exhibited metastasis nearly in all examined lymph nodes. Follow-up information was available for all 81 patients. Fifteen patients had encountered disease progression (recurrence or metastasis). Thirteen of them (86.7%, 13/15) presented lung, bone, brain, liver or upper supraclavicular lymph nodes metastases with postsurgical intervals of 1 to 111 months (mean of 35 months, and median of 12 months) and three of them (3/13) exhibited chest wall recurrence simultaneously. Two patients (13.3%, 2/15) encountered chest wall recurrence 5 and 10 months after their initial surgery, respectively. Five patients (6.2%, 5/81) died. The overall survival time ranged from 16 to 144 months, with a mean and median of 39 and 32 months.

The remaining six patients of Group 2 were in disease progression of breast cancer. Four patients were metastatic breast cancers and three of them died. The overall survival time was 28 months, 34 months and 111 months, respectively. The only one who is alive had survived for 188 months. The other two patients of Group 2 were in the state of recurrence of breast cancer. They are alive and survived for 34 months and 89 months, respectively.

## Statistical analysis results

### Correlation of clinico-pathological features with average HER2 signals, average CEP17 signals and HER2/CEP17 ratio of group 2 tumors

We performed the χ^2^ analyses on the 99 Group 2 tumors to evaluate the correlation between the clinical-pathological characteristics and the average HER2 signals, average CEP17 signals and HER2/CEP17 ratio (Supplementary Table 1). The cutoff of average HER2 signals, average CEP17 signals and HER2/CEP17 ratio were defined as 3.3, 1.4 and 2.5, respectively. Patients with average HER2 signals greater than 3.3 were younger than those with average HER2 signals less than 3.3 (*P = .*04). More than 3 lymph node metastasis occurred more common for patients with HER2/CEP17 ratio > 2.5 than those with HER2/CEP17 ratio ≤ 2.5 (*P = .*05). The relationship between other features (e.g. age, histological grade, pathologic stage, etc.) and average HER2 signals, average CEP17 signals and HER2/CEP17 ratio was not statistically significant.

### Correlation of clinico-pathological features between group 2 and group 5

**Fig. 3:**
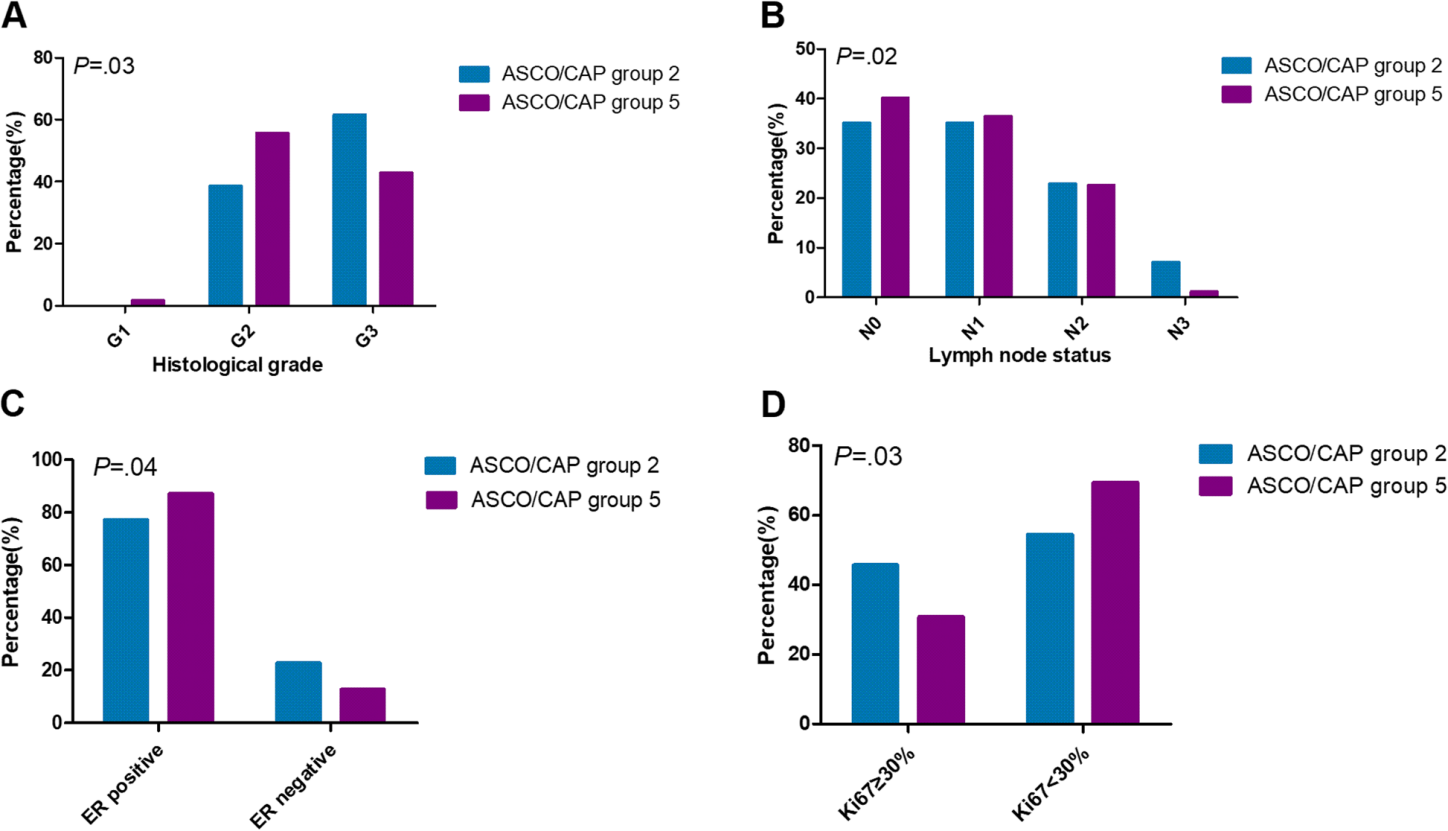
Differences in clinical pathology parameters between ASCO/CAP group 2 and ASCO/CAP group 5. **A** The proportion of grade 3 in ASCO/CAP group 2 was significantly higher than that of ASCO/CAP group 5 (*P = .*03), while no case of grade 1 was found in ASCO/CAP group 2. **B** Compared to ASCO/CAP group 5, ASCO/CAP group 2 showed much higher incidence of N3 (> 9 lymph node metastasis) (*P = .*02). **C** ER positivity was more common to seen in ASCO/CAP group 5 than that in ASCO/CAP group 2 (*P = .*04). **D** Nearly half (45.6%) patients of ASCO/CAP group 2 displayed high Ki67 level (> 30%). The proportion was significantly higher than that of ASCO/CAP group 5 (*P = .*03)

Analysis of the correlation of clinical-pathological features between 374 patients of Group 5 and 57 primary breast cancer patients of Group 2 who underwent modified radical mastectomy without neoadjuvant therapy were conducted and summarized in Table 2. Histological grade, lymph node metastasis, ER expression and Ki67 level had significant difference between the two groups (Fig. 3A-D). Compared to HER2 classical non-amplification tumors (ASCO/CAP Group 5), patients of Group 2 exhibited higher histological grade than those of Group 5 (*P = .*03). More than 9 lymph nodes metastasis was more common to be seen in Group 2 tumors than in Group 5 tumors (7.0% vs. 1.1%, *P =* .02). Positive ratio of ER was lower in Group 2 tumors than in Group 5 tumors (77.2% vs 87.2%, *P =* .04). Group 2 tumors exhibited higher ratio of Ki67 high level than Group 5 tumors (45.6% vs 30.7%, *P =* .03). Twelve patients of Group 2 were of TNBC subtype (21.1%, 12/57). The TNBC ratio in Group 5 was much lower (12.3%, 46/374), though the difference was not statistically significant (*P = .*13).

### Survival analysis of clinico-pathological parameters of ASCO/CAP group 2 patients

**Fig. 4:**
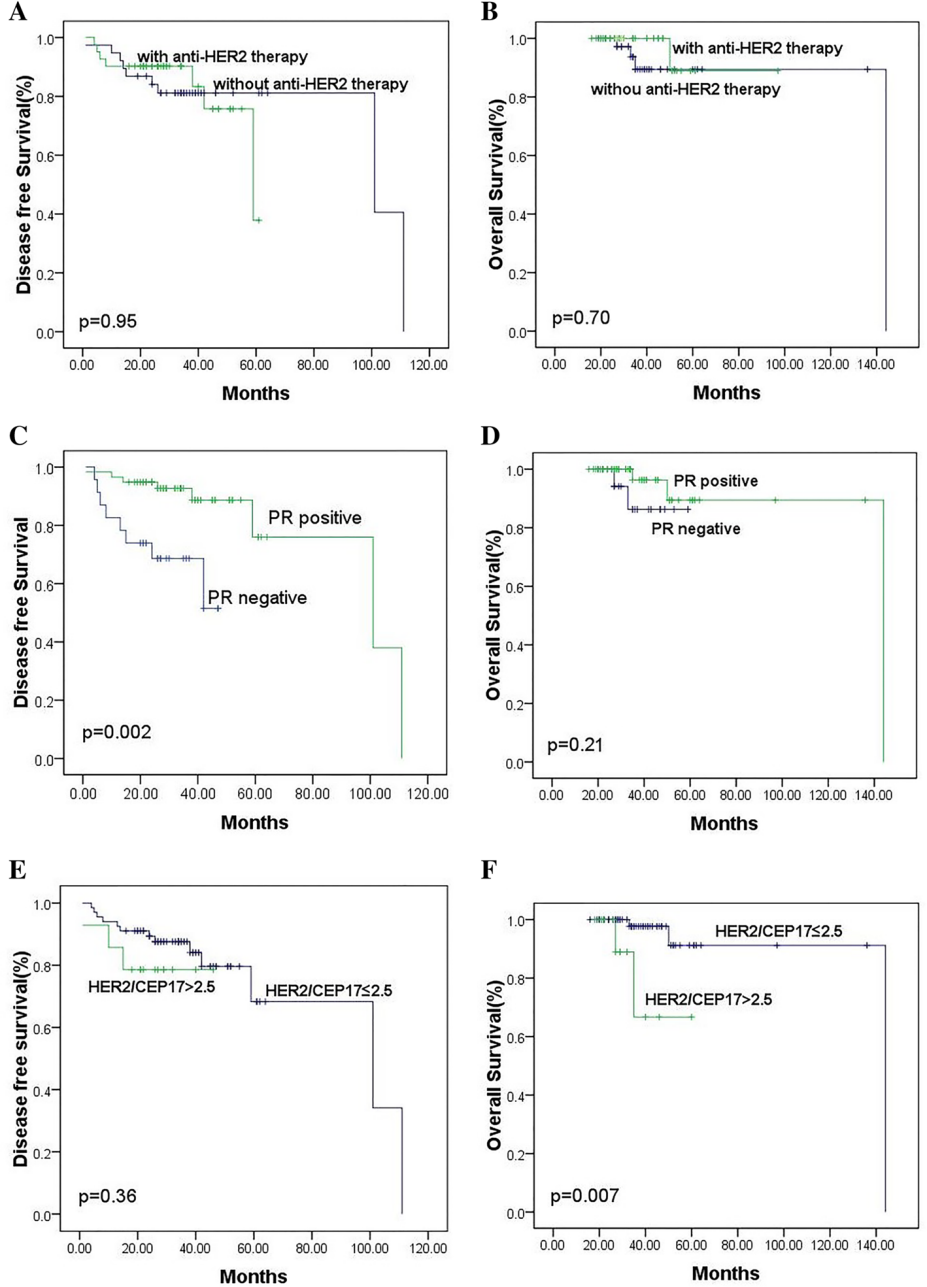
Kaplan-Meier analysis of DFS and OS of 81 ASCO/CAP group 2 primary breast cancer without neoadjuvant therapy. Patients of ASCO/CAP group 2 did not obtain apparent benefit from anti-HER2 therapy, either in terms of DFS (**A**, *P = .*95) or OS (**B**, *P* = .70). DFS of patients with PR positive was superior to that of patients with PR negative (C, *P = .*002). Patients with ratio of HER2/CEP17 ≤ 2.5 displayed a significantly better OS than those with ratio of HER2/CEP17 > 2.5 (F, *P = .*007)

We performed Kaplan–Meier analyses to evaluate the clinico-pathological features on outcomes of 81 primary breast cancers without neoadjuvant therapy prior to surgery (Supplementary Table 2). Overall survival of patients with ratio of HER2/CEP17 ≤ 2.5 was superior to that of patients with HER2/CEP17 > 2.5 (*P = .*007, Fig. 4F). The difference in DFS between the two groups was not statistically significant (*P = .*36, Fig. 4E). Patients with PR positive displayed a significantly better DFS than those with PR negative (*P = .*002) (Fig. 4C). There is no significant survival differences between patients with PR positive and those with PR negative in terms of OS (*P = .*21, Fig. 4D). Survival of patients with ER positive seemed better than that of patients with ER negative, but it was not statistically significant (*P = .*05(DFS and OS)). Difference between other clinico-pathological features and survival did not reach statistically significant.

### Correlation of anti-HER2 therapy with patients’ prognosis and survival analysis

Usage of anti-HER2 therapy was known for 79 patients of the 81 cases of primary breast cancer without neoadjuvant therapy. Forty-one patients (50.6%, 41/81) were primary breast cancer received anti-HER2 therapy after initial surgery. Six of them (14.6%, 6/41) exhibited disease progression and 1 (1/6) died. Of the other 38 patients that without anti-HER2 therapy, 9 patients (23.7%, 9/38) showed disease progression and 4 (10.5%, 4/38) of them died. Treatment after surgery of two patients was unknown for out of contact.

Of the 12 patients with neoadjuvant therapy prior to surgery, 7 (58.3%, 7/12) of them received anti-HER2 neoadjuvant therapy. One (14.3%. 1/7) of them died. None of them exhibited disease progression. Of the 5 patients without anti-HER2 neoadjuvant therapy, 2 patients (40%, 2/5) died and occurred disease progression.

Of the 6 patients who were in stage of metastasis or recurrence of breast cancer, 4 (66.7%, 4/6) of them received anti-HER2 neoadjuvant therapy. Two of them (50.0%, 2/4) died with other metastasis. The other two patients without anti-HER2 were still alive.

The correlation between anti-HER2 therapy and the prognosis of patients was not statistically significant (Supplementary Table 3). We performed further Kaplan–Meier analyses to evaluate the potential influence of anti-HER2 therapy on outcomes of 81 primary breast cancers without neoadjuvant therapy prior to surgery. The 5-year DFS of patients with anti-HER2 therapy and that of patients without anti-HER2 therapy is 36.8, 81.8%, respectively. The 5-year OS of the above two groups were almost the same (88.9% VS 88.4%). The present study did not disclose significant survival differences between patients with anti-HER2 therapy and patients without anti-HER2 therapy either in terms of DFS (*P* = .95) or OS (*P* = .70) (Supplementary Table 3, Fig. 4A and B).

## Discussion

The determination of HER2 status is of the utmost importance for breast cancer patients, for the reason of impressive improved clinical outcome of patients who received anti-ERBB2 therapies in metastatic and adjuvant settings [16–18], as well as in the neoadjuvant setting [19, 20]. Currently, the dual-probe fluorescence in situ hybridization is a validated and well-accepted method for ERBB2 testing. HER2 gene status is evaluated by determining the HER2 gene copies per chromosome 17 centromere. However, HER2/CEP17 ratio as a diagnostic criterion is influenced by the copy number of chromosome 17 and the impact of this on treatment decisions is unknown. Abnormalities in chromosome 17 are common in breast cancer with monosomy 17 reported to be much less than polysomy 17 [21].

It is not entirely clear that breast cancers of Group 2 are definitely amplified in a biologically relevant manner, or whether this finding is merely an artifact of testing methodology and interpretation. The HER2 FISH results frequently occur in the setting of true monosomy (loss of a chromosome 17), a portion loss of chromosome 17, or genetic alterations that impair CEP17 binding [8]. Patients of Group 2 even have been defined as monosomy 17 directly in previous study [22]. Due to the differences in sample size, types of materials examined, case selection criteria and methods used to define monosomy, the prevalence for monosomy 17 varied greatly in previous studies (0–38%) [23–32]. The exact overall incidence of monosomy 17 is actually unknown and few data on monosomy are presented in the literature. Breast cancer of Group 2 is rare with reported incidence of about 0.4–1.4% [6, 33–37]. In our study, 67 patients (0.8%) from 8544 in-house breast cancer patients with FISH results were enrolled. There are very few data on the clinicopathological characteristics of the breast cancers patients of Group 2 due to the low incidence.

Compared to the previous study [6], patients of Group 2 in our study exhibited higher histological grade, higher lymph node positive rate, and lager tumor size. More than half (65.4%) of our patients were of high histological grade, which is 22% higher than the reported [6]. The percentage (49.4%, 38/77) of lymph node metastases in our study was also higher than the reported (38.5%) [6]. There was no low grade patient in our study. While low grade was seen in up to 14.2% patients of other study the reported [6]. These discrepancies may be explained by two main factors: (1) the bias of patient selection. We only enrolled patients with both FISH and IHC results in our study, and most of them were HER2 IHC 2+. That is to say, a considerable number of patients with HER2 IHC negative (0/1+) were not included in our study. Whereas patients with higher HER2 protein expression was reported to be associated with poor prognosis indictors [18]. (2) Different number of patients. We included 99 cases, almost five times the number of patients in the previous study [6].

The ASCO/CAP recommendation updated in 2018 redefined the interpretation criteria in order to achieve the most accurate HER2 status identification (positive or negative) based on the conjunction of IHC and FISH results. The final HER2 status of Group 2 cases would be highly dependent on the IHC results. Except HER2 IHC 3+, both HER2 IHC negative (0/1+) and equivocal (2+) cases would be classified as HER2 negative. However, patients of this group showed HER2 IHC negative or equivocal with IHC negative cases dominantly [6, 7, 34–36]. In our study, 25 (25.3%, 25/99) primary breast cancers of Group 2 were IHC negative. All the remaining (74.7%, 74/99) exhibited IHC 2+. The proportion of IHC 2+ in our study was much higher than reported in previous reports [6, 7, 34–36]. That is because the patients with IHC 2+ account for the majority of patients who received FISH testing in our institution. Given that IHC3+ is extremely rare in this group of patients, if encountered, you need to be alert to false positive or misinterpretation. Based on the above-mentioned explanation, patients meeting Group 2 criteria were mainly HER2 non-amplification.

There is still controversy regarding the efficacy of anti-HER2 therapy in Group 2 patients. The 2013 ASCO/CAP Guideline considered these cases as HER2 amplified based on limited data from the HERA trials, which found that patients with HER2/CEP17 ratio ≥ 2.0 and average HER2 signals per cell less than 4.0 did not appear to show non-responsiveness for trastuzumab [38]. However, in the N9831 trastuzumab trial, patients with HER2/CEP17 ratio greater than 2.0 and immunochemically negative did not receive definite benefit from treatment with trastuzumab [39]. Similar result could also be seen in the BCIRG-006 clinical trial, where patients of Group 2 did not obtain apparent benefit from trastuzumab therapy, either in terms of DFS or in terms of OS [35]. Other studies reported a reduced response rate to anti-HER2 therapies, but no perceptible effect on the progression time [6]. A recent study [8] which defined HER2-amplified breast cancers with m17 as CEP17 signals < 1.5 per nucleus and HER2/CEP17 ratio ≥ 2.0 showed that outcomes of such trastuzumab-treated patients was similar to other HER2 positive patients. However, this study did not specifically evaluate the patients with average HER2 copy number less than 4. In our study, patients of Group 2 did not derive improvement from anti-HER2 therapy either in terms of DFS or in terms of OS, which was concordant with previous reports [6, 35, 39, 40]. The fact that Group2 patients showed poor response to anti-HER2 therapy might be attributed to the high incidence of monosomy in this group. First, in addition to the HER2 gene, chromosome 17 contains other genes that are essential to breast cancer pathogenesis and DNA repair, including breast cancer 1 (BRCA1), topoisomerase II-a (TOP2A), tumor protein P53 (TP53), etc. [41]. As a result, loss of chromosome 17 may have undesirable biologic effects on the therapeutic response. For instance, TOP2A had been proved associated with anthracycline sensitivity and therefore, loss of TOP2A may mediate chemoresistance and then weaken the therapeutic response [42, 43]. Second, in fact, the HER2 copy number of Group 2 tumors may not increase. In particular, if there is no true HER2 amplification, genetic alterations that impair CEP17 binding may result in a false increase in the HER2/CEP17 ratio. In such case, these tumors may not respond to anti-HER2 treatment [8]. Due to the extremely low incidence, there were too few clinical trials for patients of Group 2. Although most studies suggest that this group of patients did not gain apparent benefits from anti-HER2 treatment, it’s still too early to draw definitive conclusions whether the latter is effective or not. Meanwhile, in the recent NCT02564900 clinical trial, trastuzumab deruxtecan (DS-8201a) demonstrated promising preliminary antitumor activity in patients with HER2-low breast cancer [44]. With HER2-low expression tumors, patients of Group 2 could receive promising therapeutic effects from DS-8201a. Further researches are needed in the future.

In this study, we compared the clinicopathological characteristics of patients of Group 2 and Group 5 (i.e. classic HER2-nonamplified cancer with HER2/CEP17 ratio less than 2.0) to determine whether there is a significant difference between these two groups. We found that the average HER2 signals and the proportion of m17 were significantly higher in Group 2 patients than in Group 5 patients (*p* < 0.0001). Besides, compared to patients of Group 5, those of Group 2 exhibited higher proportion of histological grade 3 (*P = .*03) and high ki67 level (*P = .*03). More than 9 lymph node metastasis was more common in Group 2 than in Group 5 (*P = .*02). The ER positivity was lower in Group 2 than in Group 5 (*P = .*04). Compared with the classic HER2-nonamplified group, patients in Group 2 seem to have different biological behaviors. We strongly recommend that this group still be considered as a separate category, leaving the possibility to accumulate more cases and expand the scope of research in the future.

In summary, patients of ASCO/CAP Group 2 are extremely rare. To our knowledge, this is by far the largest cohort. The efficacy of anti-HER2 therapy for this group is still controversial. Even though most studies suggest that patients of this group did not gain apparent benefits from anti-HER2 treatment, it’s still too early to draw definitive conclusions whether anti-HER2 treatment was effective or not for Group 2 patients. Although HER2 is predominantly negative in patients of this group according to the updated guidelines and current reports, they exhibited higher rates of grade 3, pN3, high Ki67 level, and lower ER positivity when compared to classic HER2-nonamplified cancers. We suggest that this group should still be considered as a separate category, leaving the possibility to accumulate more cases and expand the scope of research in the future.

## Supplementary Information

**Additional file 1: Supplementary Table 1.** Correlation of clinico-pathological characteristics with average HER2 signals, average CEP17 signals and ratio of HER2/CEP17 of ASCO/CAP group 2. **Supplementary Table 2.** Kaplan–Meier analyses of 81 ASCO/CAP Group 2 primary breast cancers without neoadjuvant therapy prior to surgery. **Supplementary Table 3.** Correlation between anti-HER2 therapy and disease progression and survival of ASCO/CAP Group 2.

## Data Availability

The datasets generated during and analyzed during the current study are not publicly available due to patient privacy reasons but are available from the corresponding author on reasonable request.

## Abbreviations

ASCO/CAP: American Society of Clinical Oncology and the College of American
IHC: Immunohistochemical
HER2: Human epidermal growth factor receptor 2
m17: Monosomy of chromosome 17
MP: Miller-Payne
ER: Estrogen receptor
PR: Progesterone receptor
FISH: Fluorescence in situ hybridization
DFS: Disease free survival
OS: Overall survival
TNBC: Triple negative breast cancer

## References

[1] Slamon DJ, Clark GM, Wong SG, Levin WJ, Ullrich A, McGuire WL (1987). Human breast cancer: correlation of relapse and survival with amplification of the HER-2/neu oncogene. Science.

[2] Press MF, Bernstein L, Thomas PA, Meisner LF, Zhou JY, Ma Y (1997). HER-2/neu gene amplification characterized by fluorescence in situ hybridization: poor prognosis in node-negative breast carcinomas. J Clin Oncol.

[3] Wolff AC, Hammond ME, Schwartz JN, Schwartz JN, Hagerty KL, Allred DC (2007). American Society of Clinical Oncology/College of American Pathologists guideline recommendations for human epidermal growth factor receptor 2 testing in breast cancer. J Clin Oncol.

[4] Wolff AC, Hammond ME, Hicks DG, Dowsett M, McShane LM, Allison KH (2013). Recommendations for human epidermal growth factor receptor 2 testing in breast cancer: American Society of Clinical Oncology/College of American Pathologists clinical practice guideline update. J Clin Oncol.

[5] Wolff AC, Hammond M, Allison KH, Harvey BE, Mangu PB, Bartlett JM (2018). Human epidermal growth factor receptor 2 testing in breast Cancer: American Society of Clinical Oncology/College of American Pathologists Clinical Practice Guideline Focused Update. J Clin Oncol.

[6] Zare SY, Lin L, Alghamdi AG, Daehne S, Roma AA, Hasteh F (2019). Breast cancers with a HER2/CEP17 ratio of 2.0 or greater and an average HER2 copy number of less than 4.0 per cell: frequency, immunohistochemical correlation, and clinicopathological features. Hum Pathol.

[7] Liu ZH, Wang K, Lin DY, Xu J, Chen J, Long XY (2019). Impact of the updated 2018 ASCO/CAP guidelines on HER2 FISH testing in invasive breast cancer: a retrospective study of HER2 fish results of 2233 cases. Breast Cancer Res Treat.

[8] Page DB, Wen H, Brogi E, Dure D, Ross D, Spinelli KJ (2018). Monosomy 17 in potentially curable HER2-amplified breast cancer: prognostic and predictive impact. Breast Cancer Res Treat.

[9] Risio M, Casorzo L, Redana S, Montemurro F (2005). HER2 gene-amplified breast cancers with monosomy of chromosome 17 are poorly responsive to trastuzumab-based treatment. Oncol Rep.

[10] Gabriel N, Hortobagyi JLC, Amin MB (2016). AJCC Cancer Staging Manual. AJCC Cancer Staging Manua.

[11] Lakhani SR, Ellis LO, Schnitt SJ, Tan PH, van de Vijiver MJ (2012). WHO classification of tumors of the breast.

[12] Bloom HJ, Richardson WW (1957). Histological grading and prognosis in breast cancer; a study of 1409 cases of which 359 have been followed for 15 years. Br J Cancer.

[13] Ogston KN, Miller ID, Payne S, Hutcheon AW, Sarkar TK, Smith I (2003). A new histological grading system to assess response of breast cancers to primary chemotherapy: prognostic significance and survival. Breast.

[14] Hammond ME, Hayes DF, Dowsett M, Allred DC, Hagerty KL, Badve S (2010). American Society of Clinical Oncology/college of American pathologists guideline recommendations for immunohistochemical testing of estrogen and progesterone receptors in breast cancer. J Clin Oncol.

[15] Goldhirsch A, Winer EP, Coates AS, Gelber RD, Gebhart MP, Thürlimann B (2013). Personalizing the treatment of women with early breast cancer: highlights of the St Gallen international expert consensus on the primary therapy of early breast Cancer 2013. Ann Oncol.

[16] Pai T, Shet T, Patil A, Shetty M, Singh A, Desai SB (2018). Utility of alternate, Noncentromeric chromosome 17 reference probe for human epidermal growth factor receptor fluorescence in situ hybridization testing in breast Cancer cases. Arch Pathol Lab Med.

[17] Vogel CL, Cobleigh MA, Tripathy D, Gutheil JC, Harris LN, Fehrenbacher L (2002). Efficacy and safety of trastuzumab as a single agent in first-line treatment of HER2-overexpressing metastatic breast cancer. J Clin Oncol.

[18] Romond EH, Perez EA, Bryant J, Suman VJ, Geyer CE, Davidson NE (2005). Trastuzumab plus adjuvant chemotherapy for operable HER2-positive breast cancer. N Engl J Med.

[19] Kokate P, Sawaimoon S, Bhatia S, Mandava S (2012). Evaluation of genetic status of HER-2/neu and aneusomy 17 by fluorescence in situ hybridization and comparison with immunohistochemistry assay from Indian breast cancer patients. Genet Test Mol Biomarkers.

[20] Brunelli M, Nottegar A, Bogina G, Caliò A, Cima L, Eccher A (2015). Monosomy of chromosome 17 in breast cancer during interpretation of HER2 gene amplification. Am J Cancer Res.

[21] Tubbs RR, Pettay JD, Roche PC, Stoler MH, Jenkins RB, Grogan TM (2001). Discrepancies in clinical laboratory testing of eligibility for trastuzumab therapy: apparent immunohistochemical false-positives do not get the message. J Clin Oncol.

[22] Jimenez RE, Wallis T, Tabasczka P, Visscher DW (2000). Determination of her-2/Neu status in breast carcinoma: comparative analysis of immunohistochemistry and fluorescent in situ hybridization. Mod Pathol.

[23] Takehisa M, Sasa M, Bando Y, Hirose T, Morimoto T, Nagao T (2007). Chromosomal aneusomy (chr 1, 11, 17) detected by fluorescence in situ hybridization may be a prognostic factor in breast cancer. Anticancer Res.

[24] Merola R, Mottolese M, Orlandi G, Vico E, Cognetti F, Sperduti I (2006). Analysis of aneusomy level and HER-2 gene copy number and their effect on amplification rate in breast cancer specimens read as 2+ in immunohistochemical analysis. Eur J Cancer.

[25] Watters AD, Going JJ, Cooke TG, Bartlett JM (2003). Chromosome 17 aneusomy is associated with poor prognostic factors in invasive breast carcinoma. Breast Cancer Res Treat.

[26] Wang S, Hossein SM, Frenkel EP, Haley BB, Siddiqui MT, Gokaslan S (2002). Aneusomy 17 in breast cancer: its role in HER-2/neu protein expression and implication for clinical assessment of HER-2/neu status. Mod Pathol.

[27] Nakopoulou L, Giannopoulou I, Trafalis D, Trafalis D, Gakiopoulou H, Keramopoulos A (2002). Evaluation of numeric alterations of chromosomes 1 and 17 by in situ hybridization in invasive breast carcinoma with clinicopathologic parameters. Appl Immunohistochem Mol Morphol.

[28] Fehm T, Morrison L, Saboorian H, Hynan L, Tucker T, Uhr J (2002). Patterns of aneusomy for three chromosomes in individual cells from breast cancer tumors. Breast Cancer Res Treat.

[29] Botti C, Pescatore B, Mottolese M, Sciarretta F, Greco C, Filippo FD (2000). Incidence of chromosomes 1 and 17 aneusomy in breast cancer and adjacent tissue: an interphase cytogenetic study. J Am Coll Surg.

[30] Reinholz MM, Bruzek AK, Visscher DW, Lingle WL, Schroeder MJ, Perez EA (2009). Breast cancer and aneusomy 17: implications for carcinogenesis and therapeutic response. Lancet Oncol.

[31] Buzdar AU, Valero V, Ibrahim NK, Francis D, Broglio KR, Theriault RL (2007). Neoadjuvant therapy with paclitaxel followed by 5-fluorouracil, epirubicin, and cyclophosphamide chemotherapy and concurrent trastuzumab in human epidermal growth factor receptor 2-positive operable breast cancer: an update of the initial randomized study population and data of additional patients treated with the same regimen. Clin Cancer Res.

[32] Buzdar AU, Ibrahim NK, Francis D, Booser DJ, Thomas ES, Theriault RL (2005). Significantly higher pathologic complete remission rate after neoadjuvant therapy with trastuzumab, paclitaxel, and epirubicin chemotherapy: results of a randomized trial in human epidermal growth factor receptor 2-positive operable breast cancer. J Clin Oncol.

[33] Ballard M, Jalikis F, Krings G, Schmidt RA, Chen YY, Rendi MH (2017). ‘Non-classical’ HER2 FISH results in breast cancer: a multi-institutional study. Mod Pathol.

[34] Press MF, Villalobos I, Santiago A, Guzman R, Cervantes M, Gasparyan A (2016). Assessing the new American Society of Clinical Oncology/College of American Pathologists Guidelines for HER2 testing by fluorescence in situ hybridization: experience of an academic consultation practice. Arch Pathol Lab Med.

[35] Press MF, Sauter G, Buyse M, Fourmanoir H, Quinaux E, Tsao-Wei DD (2016). HER2 gene amplification testing by fluorescent in situ hybridization (FISH): comparison of the ASCO-College of American pathologists guidelines with FISH scores used for enrollment in breast Cancer international research group clinical trials. J Clin Oncol.

[36] Shah MV, Wiktor AE, Meyer RG, Tenner KS, Ballman KV, Green SJ (2016). Change in pattern of HER2 fluorescent in situ hybridization (FISH) results in breast cancers submitted for FISH testing: experience of a reference laboratory using US Food and Drug Administration criteria and American Society of Clinical Oncology and College of American Pathologists Guidelines. J Clin Oncol.

[37] Stoss OC, Scheel A, Nagelmeier I, Schildhaus HU, Henkel T, Viale G (2015). Impact of updated HER2 testing guidelines in breast cancer--re-evaluation of HERA trial fluorescence in situ hybridization data. Mod Pathol.

[38] Dowsett M, Procter M, McCaskill-Stevens W, de Azambuja E, Dafni U, Rueschoff J (2009). Disease-free survival according to degree of HER2 amplification for patients treated with adjuvant chemotherapy with or without 1 year of trastuzumab: the HERA trial. J Clin Oncol.

[39] Perez EA, Reinholz MM, Hillman DW, Tenner KS, Schroeder MJ, Davidson NE (2010). HER2 and chromosome 17 effect on patient outcome in the N9831 adjuvant trastuzumab trial. J Clin Oncol.

[40] Xl W, Teng XD, Ding W, Sun K, Wang B (2020). A clinicopathological study of 30 breast cancer cases with a HER2/CEP17 ratio of ≥2.0 but an average HER2 copy number of <4.0 signals per cell. Mod Pathol.

[41] Bieche I, Tomasetto C, Regnier CH, Moog-Lutz C, Rio MC, Lidereau R (1996). Two distinct amplified regions at 17q11-q21 involved in human primary breast cancer. Cancer Res.

[42] Engstrom MJ, Ytterhus B, Vatten LJ, Opdahl S, Bofin AM (2014). TOP2A gene copy number change in breast cancer. J Clin Pathol.

[43] Press MF, Sauter G, Buyse M, Bernstein L, Guzman R, Santiago A (2011). Alteration of topoisomerase II-alpha gene in human breast cancer: association with responsiveness to anthracycline-based chemotherapy. J Clin Oncol.

[44] Modi S, Park H, Murthy RK, Iwata H, Tamura K, Tsurutani J (2020). Antitumor activity and safety of Trastuzumab Deruxtecan in patients with HER2-low-expressing advanced breast Cancer: results from a phase Ib study. J Clin Oncol.

