# Supplementary material for: A clinicopathological study and survival analysis of 99 breast cancers with HER2/CEP17 ratio ≥ 2.0 and an average HER2 copy number < 4.0 per cell in China
Source: BMC Cancer. 2023 Jan 25;23:84. doi: 10.1186/s12885-023-10531-z (PMC9875391; doi:10.1186/s12885-023-10531-z)
Supplement: Supplementary file 1 — Additional file 1: Supplementary Table 1. Correlation of clinico-pathological characteristics with average HER2 signals, average CEP17 signals and ratio of HER2/CEP17 of ASCO/CAP group 2. Supplementary Table 2. Kaplan–Meier analyses of 81 ASCO/CAP Group 2 primary breast cancers without neoadjuvant therapy prior to surgery. Supplementary Table 3. Correlation between anti-HER2 therapy and disease progression and survival of ASCO/CAP Group 2. [file 12885_2023_10531_MOESM1_ESM.docx]

Supplementary Table 1 Correlation of clinico-pathological characteristics with average HER2 signals, average CEP17 signals and ratio of HER2/CEP17 of ASCO/CAP group 2

| Characteristics | HER2/cell | | p | CEP17/cell | | p | HER2/CEP17 | | p |
| --- | --- | --- | --- | --- | --- | --- | --- | --- | --- |
|  | 2.2-3.3 | 3.3-4.0 |  | 1-1.4 | 1.4-1.84 |  | 2.0-2.5 | 2.5-3.41 |  |
| Age(year) |  |  | 0.042 |  |  | 0.256 |  |  | 0.377 |
| <50 | 15(16.1%) | 28(30.1%) |  | 19(20.4%) | 24(25.8%) |  | 34(36.6%) | 9(9.7%) |  |
| >50 | 28(30.1%) | 22(23.7%) |  | 28(30.1%) | 22(23.7%) |  | 43(46.2%) | 7(7.5%) |  |
| Histologic grade |  |  | 0.822 |  |  | 0.186 |  |  | 0.921 |
| G2 | 13(16.0%) | 15(18.5%) |  | 17(21.1%) | 11(13.6%) |  | 23(28.4%) | 5(6.2%) |  |
| G3 | 26(32.1%) | 27(33.3%) |  | 24(29.6%) | 29(35.83%) |  | 44(65.7%) | 9(11.1%) |  |
| Pathologic stage |  |  | 0.453 |  |  | 0.17 |  |  | 0.39 |
| T1 | 20(24.7%) | 17(21.0%) |  | 22(27.2%) | 15(18.5%) |  | 33(40.7%) | 4(4.9%) |  |
| T2 | 14(17.3%) | 20(24.7%) |  | 13(16.0%) | 21(25.9%) |  | 27(33.3%) | 7(8.6%) |  |
| T3 | 2(2.5%) | 1(1.2%) |  | 2(2.5%) | 1(1.2%) |  | 3(3.7%) | 0 |  |
| Non-available | 3(3.7%) | 4(4.9%) |  | 4(4.9%) | 3(3.7%) |  | 4(4.9%) | 3(3.7%) |  |
| Lymph node status |  |  | 0.737 |  |  | 0.926 |  |  | 0.048 |
| pN0+pN1 | 31(38.3%) | 29(35.8%) |  | 31(38.3%) | 29(35.8%) |  | 53(65.4%) | 7(8.6%) |  |
| pN2+pN3 | 8(9.9%) | 9(11.1%) |  | 9(11.1%) | 8(9.9%) |  | 12(14.8%) | 5(6.2%) |  |
| Non-available | 0 | 4(4.9%) |  | 1(1.1%) | 3(3.7%) |  | 2(2.5%) | 2(2.5%) |  |
| AJCC staging |  |  | 0.629 |  |  | 0.207 |  |  | 0.588 |
| I | 14(17.3%) | 11(13.6%) |  | 16(19.8%) | 9(11.1%) |  | 22(27.2%) | 3(3.7%) |  |
| II | 14(17.3%) | 18(22.2%) |  | 13(16.0%) | 19(23.5%) |  | 29(35.8%) | 3(3.7%) |  |
| III | 8(9.9%) | 7(8.6%) |  | 7(8.6%) | 8(9.9%) |  | 12(14.8%) | 3(3.7%) |  |
| Non-available | 3(3.7%) | 6(7.4%) |  | 5(6.2%) | 4(4.9%) |  | 4(4.9%) | 5(6.2%) |  |
| ER |  |  | 0.255 |  |  | 0.117 |  |  | 0.335 |
| positive | 36(38.7%) | 37(39.8%) |  | 40(43.0%) | 33(35.5%) |  | 59(63.4%) | 14(15.1%) |  |
| negative | 7(7.5%) | 13(14.0%) |  | 7(7.5%) | 13(14.0%) |  | 18(19.4%) | 2(2.2%) |  |
| PR |  |  | 0.337 |  |  | 0.455 |  |  |  |
| positive | 28(30.1%) | 36(38.7%) |  | 32(34.4%) | 32(34.4%) |  | 52(55.9%) | 12(12.9%) | 0.557 |
| negative | 15(16.1%) | 14(15.1%) |  | 15(16.1%) | 14(15.1%) |  | 25(26.9%) | 4(4.3%) |  |
| HER2(IHC) |  |  | 0.238 |  |  | 0.218 |  |  | 0.292 |
| negative (0,1+) | 9(9.7%) | 16(17.2%) |  | 10(10.8%) | 15(16.1%) |  | 19(20.4%) | 6(6.5%) |  |
| equivocal (2+) | 34(36.6%) | 34(36.6%) |  | 37(39.8%) | 31(33.3%) |  | 58(62.4%) | 10(10.8%) |  |
| Ki67 |  |  | 0.961 |  |  | 0.256 |  |  | 0.203 |
| ≥30% | 20(21.5%) | 23(24.7%) |  | 19(20.4%) | 24(25.8%) |  | 38(40.9%) | 5(5.4%) |  |
| <30% | 23(24.7%) | 27(29.0%) |  | 28(30.1%) | 22(23.7%) |  | 39(41.9% | 11(11.8%) |  |

Supplementary Table 2 Kaplan–Meier analyses of 81 ASCO/CAP Group 2 primary breast cancers without neoadjuvant therapy prior to surgery

| Parameters | DFS | OS |
| --- | --- | --- |
| Age | 0.06 | 0.116 |
| Histologic grade | 0.222 | 0.397 |
| Pathologic stage | 0.506 | 0.276 |
| Lymph node status | 0.877 | 0.113 |
| AJCC staging | 0.877 | 0.061 |
| ER | 0.053 | 0.051 |
| PR | 0.002 | 0.208 |
| Ki67 level | 0.587 | 0.511 |
| Subtype | 0.19 | 0.255 |
| anti-HER2 therapy | 0.953 | 0.704 |
| HER2/cell | 0.935 | 0.521 |
| CEP17/cell | 0.936 | 0.105 |
| Ratio of HER2/CEP17 | 0.361 | 0.007 |

Supplementary Table 3 Correlation between anti-HER2 therapy and disease progression and survival of ASCO/CAP Group 2

| Patients of Group 2 | Disease progression | | p | Survival | | p |
| --- | --- | --- | --- | --- | --- | --- |
|  | progression | progression free |  | die | alive |  |
| Primary breast cancer without neoadjuvant therapy |  |  | 0.464 |  |  | 0.14 |
| anti-HER2 therapy(n=41) | 6(14.6%) | 35(85.4%) |  | 1(2.4%) | 40(97.6%) |  |
| without anti-HER2 therapy(n=38) | 9(23.7%) | 29(76.3%) |  | 4(10.5%) | 34(89.5%) |  |
| Non-available(n=2) | 0 | 2(100.0%) |  | 0 | 2(100.0%) |  |
| Primary breast cancer with neoadjuvant therapy |  |  | 0.067 |  |  | 0.31 |
| anti-HER2 therapy(n=7) | 0 | 7(100.0%) |  | 1(14.3%) | 6(85.7%) |  |
| without anti-HER2 therapy(n=5) | 2(40.0%) | 3(60.0%) |  | 2(40.0%) | 3(60.0%) |  |
| Metastasis and recurrence of breast cancer |  |  | 1.00 |  |  | 1.00 |
| anti-HER2 therapy(n=4) | 2(50.5%) | 2(50.5%) |  | 2(50.5%) | 2(50.5%) |  |
| without anti-HER2 therapy(n=2) | 1(50.0%) | 1(50.0%) |  | 1(50.0%) | 1(50.0%) |  |
